# THE PSYCHOSOCIAL IMPACT OF COVID-19 ON COGNITIVE AGING ARAB AMERICAN IMMIGRANTS IN MICHIGAN

**DOI:** 10.1093/geroni/igac059.873

**Published:** 2022-12-20

**Authors:** Linda Sayed

**Affiliations:** Michigan State University, East Lansing, Michigan, United States

## Abstract

This study examines the psychosocial impact of COVID-19 on cognitive aging Middle Eastern/Arab Americans immigrants and refugees in Michigan. Sociopolitical experiences as an immigrant and/or refugee may have unique effects on late-life cognitive health. Given that social engagement reduces the risk of ADRD, this study sought to examine the consequences of COVID-19 socialization restrictions on familial and communal support systems for aging Middle Eastern/Arab immigrants and refugees. Three focus groups discussions with 8-10 participants each (N=31) conducted at local Arab organizations and religious institutions were conducted followed by inductive analysis. Preliminary results indicate two prominent themes: 1) Social and Familial Hardships; and 2) Isolation. Narratives illustrate the prevalence of psychosocial ADRD risk from COVID-19. Findings are discussed within a stress framework to advance universal and unique elements of Arab immigrant and refugee cognitive aging.

